# Histone H1 Post-Translational Modifications: Update and Future Perspectives

**DOI:** 10.3390/ijms21165941

**Published:** 2020-08-18

**Authors:** Marta Andrés, Daniel García-Gomis, Inma Ponte, Pedro Suau, Alicia Roque

**Affiliations:** 1 Biochemistry and Molecular Biology Department, Biosciences Faculty, Autonomous University of Barcelona, 08193 Cerdanyola del Vallès, Spain; marta.andres@uab.es (M.A.); daniel.garcia.gomis@uab.es (D.G.-G.); inma.ponte@uab.es (I.P.); pere.suau@uab.es (P.S.); 2 Instituto de Biología Molecular de Barcelona (IBMB-CSIC), 08028 Barcelona, Spain

**Keywords:** histone H1, chromatin structure, PTM function, disease, phosphorylation, methylation, acetylation, ubiquitylation, citrullination, mass spectrometry

## Abstract

Histone H1 is the most variable histone and its role at the epigenetic level is less characterized than that of core histones. In vertebrates, H1 is a multigene family, which can encode up to 11 subtypes. The H1 subtype composition is different among cell types during the cell cycle and differentiation. Mass spectrometry-based proteomics has added a new layer of complexity with the identification of a large number of post-translational modifications (PTMs) in H1. In this review, we summarize histone H1 PTMs from lower eukaryotes to humans, with a particular focus on mammalian PTMs. Special emphasis is made on PTMs, whose molecular function has been described. Post-translational modifications in H1 have been associated with the regulation of chromatin structure during the cell cycle as well as transcriptional activation, DNA damage response, and cellular differentiation. Additionally, PTMs in histone H1 that have been linked to diseases such as cancer, autoimmune disorders, and viral infection are examined. Future perspectives and challenges in the profiling of histone H1 PTMs are also discussed.

## 1. Introduction

Histone H1 is a key chromatin structural protein, which mediates higher-order chromatin folding. H1 is also emerging as an important epigenetic mark and regulator of gene expression and cellular differentiation. Metazoan H1 has three structural domains: a short N-terminal domain (NTD), a central globular domain (GD), and a long C-terminal domain (CTD). The globular domain contains a winged-helix motif and is responsible for the binding of H1 to nucleosomal DNA. Both terminal domains are intrinsically disordered and have a positive net charge in physiological conditions due to the abundance of lysine residues [1,2]. The CTD is considered the main determinant of H1-driven chromatin compaction [3]. This domain is also responsible for the preference for the scaffold-associated regions (SAR-DNA) [4], for the interaction with apoptotic nuclease DFF40 [5], with the β-amyloid peptide, and the formation of amyloid-like fibers [6].

Histone H1 is the most divergent and heterogeneous group of histones. It has been suggested that the first H1-like proteins may have appeared early in evolution in eubacteria, while the sequence winged-helix motif, present in the GD, appeared much later, in protists [7]. Detection of putative recombination points suggest that this process may have been involved in the acquisition of the H1 tripartite structure [8]. In lower eukaryotes, histone H1 is very heterogeneous. Some protists, like *Tetrahymena thermophila*, are only lysine-rich with a sequence composition similar to bacterial H1-like proteins and the CTD of metazoans, while the *Saccharomyces cerevisiae* version of H1, HhoI, contains a second winged-helix motif at its C-terminal end [7,9]. However, it has been reported more recently that the high mobility group protein HMO1 functions as a linker histone in *Saccharomyces cerevisiae* [10].

In mammals, 11 H1 subtypes have been identified [11]. Subtypes H1.0–H1.5 and H1x are differentially expressed in somatic cells, while subtypes H1t, HILS1, and H1T2 are expressed in male germinal cells, and H1oo is expressed in oocytes [12]. Orthologous genes are readily identified across mammalian species, but they are rarely detected in moderately distant phyla. Mammalian H1 subtypes differ in their evolution velocities [7,13], expression patterns [12], chromatin binding affinity [14,15,16], and genomic distribution [17,18,19,20], among other features. The presence of post-translational modifications (PTMs) adds a new level of complexity to H1 diversity, as individual PTMs or their interplay can modulate protein structure and function. In this review, we present a comprehensive summary of the PTMs identified in H1, from lower eukaryotes to humans, focusing on those modifications with known biological function, or associated with disease.

### Overview of Histone H1 PTMs from Lower Eukaryotes to Humans

The first post-translational modification in H1 was described in the 70s, when phosphorylated H1 was identified in different species, throughout the tree of life. In a relatively short period, H1 phosphorylation was reported in protists such as *Physarum polycephalum* [21] and *Tetrahymena sp*. [22,23], and also in animals including *Drosophila melanogaster* [24], chicken erythrocytes [25], mammalian cell lines (rat hepatoma cells (HTC), and Chinese hamster ovary cells (CHO)) [26,27]. The identification of phosphorylation was carried out based on the changes in H1 electrophoretic mobility and on the incorporation of ^32^P and/or phosphatase treatment. At the time, mapping the phosphate groups to individual residues was still elusive, and significant amounts of the modified protein were necessary for its detection.

The development of mass spectrometry-based proteomics has represented a significant breakthrough in the identification of post-translational modifications, allowing mapping of PTMs to specific residues as well as the detection of low abundant modified species. There are three main types of proteomic strategies, depending on the molecule analyzed by mass spectrometry: top-down, middle-down, and bottom-up. Top-down proteomics analyzes intact proteins, while middle-down and bottom-up proteomics analyze large protein fragments/domains and small peptides, respectively [28]. PTM mapping and identification is mostly carried out by bottom-up proteomics. In this type of proteomic strategy, intact proteins are digested into small peptides, which are analyzed by tandem mass spectrometry (MS/MS). During MS/MS, ionized peptides are selected in the first mass analyzer, fragmented, and the m/z of the product ions is determined in the second mass analyzer. Mass differences between product ions allow for peptide sequencing and PTM assignment. The availability of mass analyzers of high mass accuracy has allowed for the distinction between quasi-isobaric PTMs, trimethylation/acetylation, and dimethylation/formylation [29,30]. 

Analysis of histone H1 PTMs by bottom-up proteomics has represented a technical challenge for two main reasons. First, H1 amino acid composition is characterized by a high content of lysine residues. Therefore, digestion with trypsin yields peptides that are relatively small and hydrophilic, which are difficult to detect by MS due to poor retention in the C18 Reverse-Phase Ion-Pairing High Performance Liquid Chromatography (RP-HPLC) column. As a result, regions with the highest density of lysine residues such as the CTD tend to have low coverage. This problem has been addressed by propionylation of amine groups in the protein (N-terminal amines, and free and monomethylated lysine ε-amino groups) before or after tryptic digestion [31,32]. This modification has significantly improved the coverage of H1 by MS.

The second challenging aspect is the presence in variable quantities of multiple subtypes or variants in higher eukaryotes. In particular, mammalian subtypes H1.1–H1.5 have more than 60% of sequence identity, and the sequence of the GD is almost identical [33]. Thus, many promiscuous peptides, matching the sequence of several subtypes, are often found in bottom-up studies. Several strategies have been used for the assignment of subtype-specific PTMs. Separation of individual subtypes before proteolytic digestion have been performed by capillary electrophoresis [34] and by 2D-electrophoresis. The latter used acetic-urea (AU) or triton-acetic-urea (TAU) electrophoresis in the first dimension, and Sodium Dodecyl Sulfate PolyAcrylamide Gel Electrophoresis (SDS-PAGE) in the second dimension [35,36,37]. Combining bottom-up and top-down proteomics has also been used to assign PTMs to individual subtypes [31,38,39,40,41,42].

Bottom-up proteomics has identified and mapped a wide variety of PTMs in several eukaryotes (summarized in Tables S1–S3). To our knowledge, at least 13 PTM types have been identified in H1 including phosphorylation, methylation, acetylation, citrullination, crotonylation, ubiquitylation, formylation, 2-hydroxyisobutyrylation, and ADP-ribosylation (parylation) [29,30,32,42,43,44,45]. In this section, a brief overview of the PTMs identified in several model organisms is made. For consistency, in all cases, the modified residue number corresponds to the mature protein, which lacks the initial methionine. Therefore, for some modifications, the position number is not the same as the number in the original reference.

**Figure 1:**
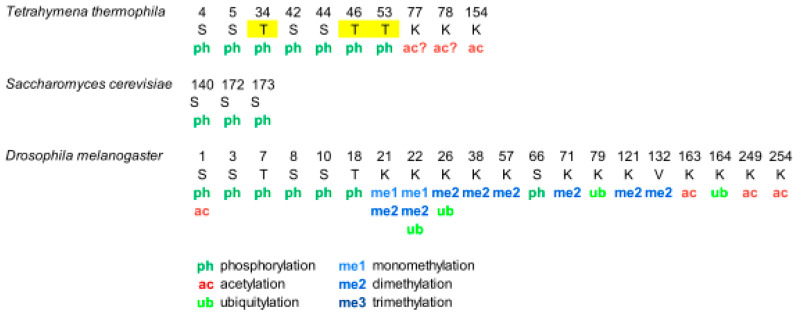
Modified positions in histone H1 of *Tetrahymena thermophila*, *Saccharomyces cerevisiae*, and *Drosophila melanogaster*. The positions refer to the mature protein, which lacks the initial methionine. Highlighted in yellow, phosphorylation in cyclin-dependent-kinase (CDK) consensus motifs. Question marks are included in post-translational modifications (PTMs) of ambiguous assignment.

Phosphorylation of histone H1 in *Tetrahymena thermophila* was detected during the 70s. However, the complete characterization of phosphorylation of macronuclear H1 by mass spectrometry, in vegetative growing cells and starved cells, was not performed until 2006 [38] (Figure 1, Table S1). This study confirmed the five phosphosites previously identified by Edman-sequencing and peptide microsequencing [46,47], which included three consensus cyclin-dependent kinases (CDK) sites with the sequence S/T-P-X-K/R: T34, T46, and T53, and two non-canonical CDK sites: S4 and S5. Additionally, the analysis identified two novel phosphorylation sites, S42 and S44, and two novel acetylation sites, K77/78 and K154 (Figure 1, Table S1).

Separation of phosphorylated species from unphosphorylated up to heptaphosphorylated H1, by cation-exchange chromatography combined with bottom-up and top-down proteomics revealed the precise hierarchy of phosphorylation in this organism, where the first phosphorylated residues corresponded to the CDK consensus motifs. Finally, relative quantification of the phosphorylated species by stable-isotope labeling, Immobilized Metal Affinity Chromatography (IMAC), and mass spectrometry showed that phosphorylated H1 was more abundant in growing *Tetrahymena* cells than in starved cells, and also confirmed the hierarchy of phosphorylation. In yeast, another unicellular eukaryote, the protein Hho I is generally considered the equivalent to metazoan histone H1. In Hho I, only three phosphorylation sites have been identified by mass spectrometry at S141, S172, and S173 (Figure 1, Table S1). The first residue, S141, is located between the two globular domains, while S172 and S173 are located in the second globular domain, WHD2 [48].

*Drosophila melanogaster* is a model organism widely used to study differentiation, control of gene expression, and several diseases [49]. In *Drosophila*, there is only one somatic linker histone (dH1), and one germ-line specific H1 (dBigH1), which is present during early embryogenesis until the zygote genome is activated [49]. The first proteomic analysis of PTMs in dH1 found modifications in the first ten amino acids during embryonic development, consisting of N-terminal acetylation, mono- and diphosphorylation (Figure 1, Table S1) [50]. Analysis of the phosphorylated positions showed that the main phosphorylation site was S10 and that the amount of this modification decreased as the embryos matured. Another four residues, S1, S3, T7, and S8 were also found phosphorylated, albeit in lower proportions than S10.

Analysis of PTMs from cultured *Drosophila* S2 cells showed the presence of additional modifications including two new phosphorylated positions T19 and S67 as well as eight methylation sites, three acetylation sites, and four ubiquitylation sites (Table S1) [39]. In some cases, more than one PTM was mapped to the same lysine residue. In this study, bottom-up proteomics was complemented with the analysis of the N-terminal domain and of the intact protein in order to determine which PTMs coexisted in the same dH1 molecule. Top-down experiments showed the existence of multiple proteoforms for dH1, containing different arrays of the PTMs identified in the bottom-up approach. Middle-down analysis of the N-terminal domain identified S8 and S10 as the positions modified in the dephosphorylated species, in agreement with the previous study [50]. Other species were detected with combinations of Nα-terminal acetylation, mono- and dephosphorylation, and dimethylation. Tri- tetra- and pentaphosphorylated species were also detected in low proportions. 

Chicken erythrocytes are a model system to study the chromatin structure [51,52,53]. In chicken erythrocytes, there are six H1 subtypes, H1.01, H1.02, H1.03, H1.10, H1.1L and H1.1R, which amount to 40% of the linker histones. H1 subtypes have more than 85% of sequence identity and lack clear orthologous with mammalian subtypes [33,54]. The high sequence identity can impair the assignment of PTMs in chicken H1 subtypes because sometimes the modified peptides are shared between several subtypes. Histone H5, which is considered orthologous to mammalian H1.0, represents the remaining 60% of the linker histones [33].

**Figure 2:**
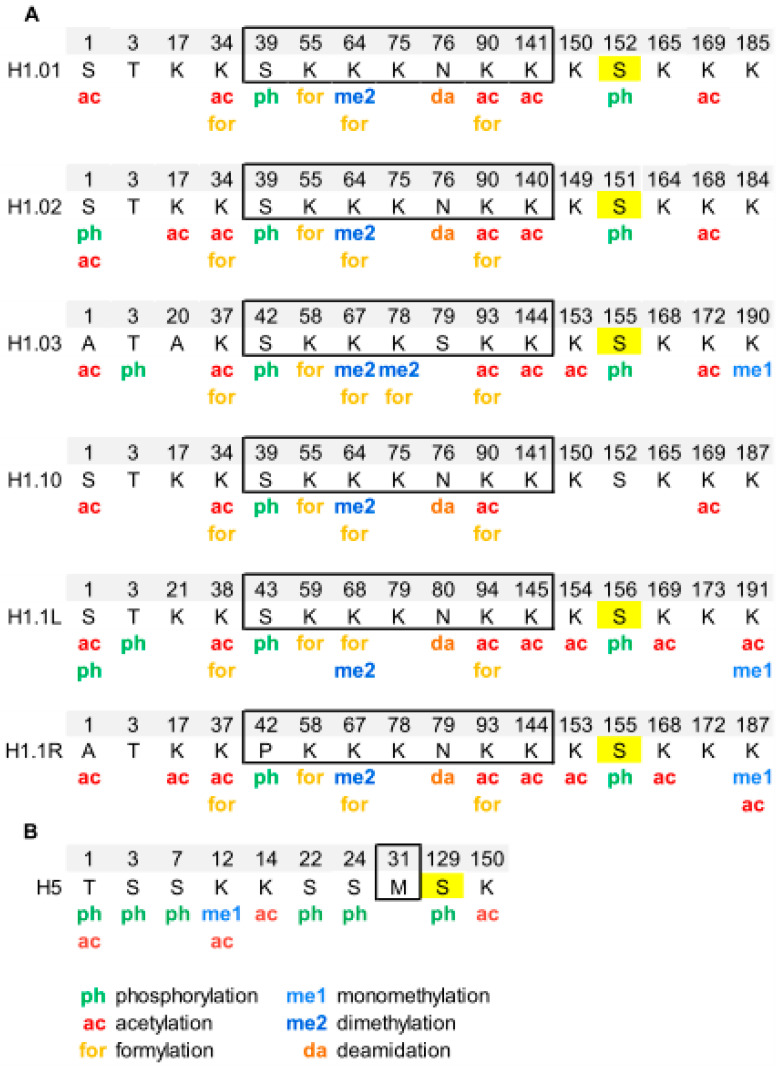
Modified positions in linker histones of chicken erythrocytes. (**A**) PTMs identified in H1 subtypes are shown in the sequence alignment. (**B**) PTMs identified in H5. The residues of the globular domain are shown inside the box. In yellow, phosphorylated residues located at CDK-consensus motifs. The positions refer to the mature protein, which lacks the initial methionine. The complete sequences and the original sequence alignment are shown in Figure S1.

Analysis of PTMs by bottom-up proteomics identified five modification types in chicken erythrocyte linker histones: acetylation, phosphorylation, methylation, formylation, and deamidation (Figure 2, Figure S1, and Table S2) [55,56]. All H1 subtypes were N-terminally acetylated. Two additional acetylated residues were found in the NTD of H1.02 and H1.1R (K17, for both subtypes). The NTD was also modified by phosphorylation in the first and/or the third residue, depending on the subtype. Two acetylations and one phosphorylation mapped to the GD, K34ac, K90ac, and S39p (referred to H1.01 sequence) were in peptides common to all H1 subtypes. The two of the acetylated sites of the GD were also found formylated. This modification was also detected in additional sites of the GD. The mass-shift caused by formylation is quasi-isobaric with that of dimethylation, thus, in some cases, the type of modification was not determined [56]. Additionally, one of the asparagine residues of the GD was found deamidated. Up to four acetylated sites were found in the CTD, depending on the subtype. Like in the GD, all the acetylated sites in this domain were found in peptides common to more than one subtype. Two monomethylated peptides belonging to the CTD were detected. One of these peptides is shared by two subtypes and was also acetylated. Finally, only one of the three CDK-consensus motifs in the CTD of H1 subtypes (S155, referred to H1.01) or in the CTD of H5 (S129) was phosphorylated. This result was expected as phosphorylation in H1 decreases during erythrocyte terminal differentiation [56]. In H5, most of the PTMs found were in the NTD. They consisted of five phosphorylations (T1, S3, S7, S22, and S24), three acetylations (T1, K12, and K14), and one monomethylation (K12). Furthermore, the CTD of H5 was phosphorylated in S129 and acetylated in K150. PTMs were differentially distributed among soluble and insoluble chromatin fractions, as shown by relative quantification [55].

**Figure 3:**
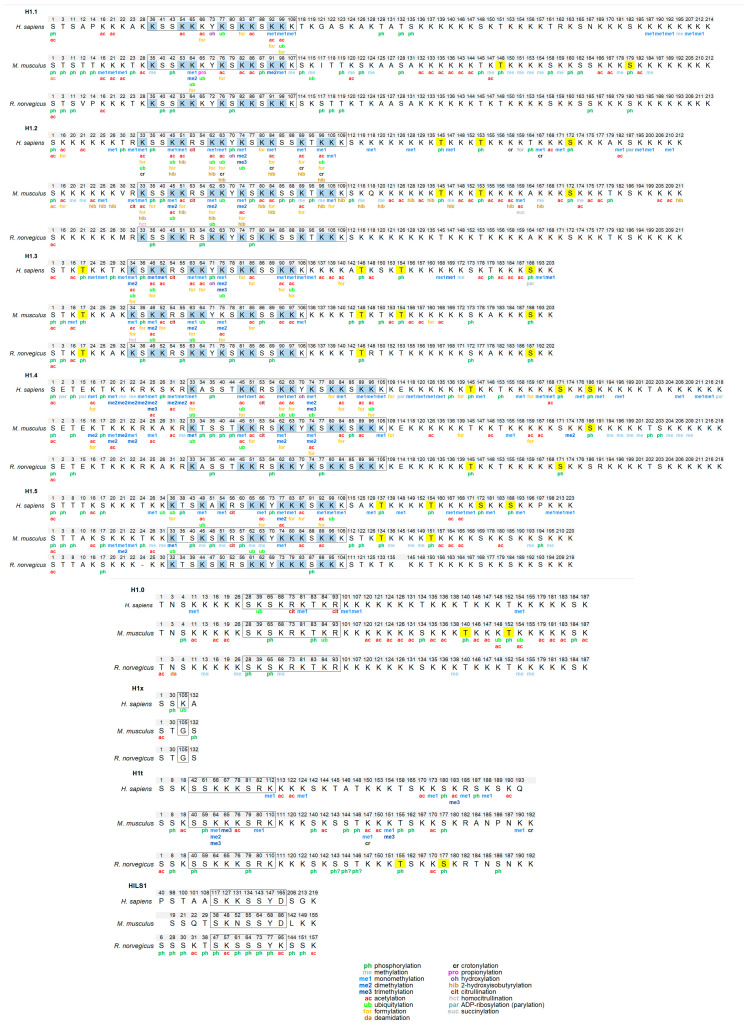
Modified positions in mammalian H1 subtypes. The PTMs are shown based on the sequence alignment of the human, mouse, and rat sequence for each subtype. The residues of the globular domain are shown in the box. In yellow, phosphorylated residues located at CDK-consensus motifs. In blue, PTM-hotspots in the globular domain. The positions refer to the mature protein, which lacks the initial methionine. The complete sequences and the original alignments are shown in Figure S2.

Identification of PTMs in histone H1 in mammals has been carried out mostly in humans and mice, although some PTMs were characterized in rat testis (Figure 3, Figure S2, Table S3). The most extensive bottom-up study identified multiple PTM types in human cell lines and several mouse tissues [29]. Other studies have targeted specific PTMs including phosphorylation [42], methylation [43], formylation [30], 2-hydroxyisobutyrylation [44], crotonylation [32] or specific cell lines [35,36], tissues [34], processes [57], and subtypes [58].

From the accumulated data, several conclusions about the abundance, distribution of PTM types, and modification types can be drawn. All somatic subtypes are post-translationally modified. However, the number of modified positions appears to be determined by the abundance of each subtype. Therefore, many PTMs have been identified in the most abundant subtypes H1.2 and H1.4, while very little information is available for low abundance subtypes such as H1.0 and H1x. H1.1 has very restricted expression, but PTM mapping has been performed in the testis and in mouse embryonic stem cells (mESCs), where this subtype is present in significant proportions [59]. In some cases, PTMs in subtypes with high sequence identity, especially in the GD, like H1.2–H1.5 and to a lesser extent H1.1, might be overestimated. However, modifications in the terminal domains are often subtype-specific, as the sequence identity between subtypes is much lower in the terminal domains [33].

Despite the fact that new PTM types like formylation, crotonylation. or citrullination have been described, the most widespread modifications in histone H1 are phosphorylation, methylation, and acetylation. H1 subtypes contain between 27–44 residues that can be phosphorylated, of which up to 40% of them have been found to be phosphorylated (Table S4). Phosphorylated positions have been detected in all structural domains, but most of them are in the terminal domains. The NTD of subtypes H1.1–H1.5 contains two phosphorylation hotspots, the SET motif, and a CDK consensus motif or another residue phosphorylated during the cell cycle. The rest of the CDK consensus motifs (three or four, depending on the subtype) are in the CTD. Phosphorylation is highly abundant in H1, as up to 75% of the proteins are phosphorylated in mitosis [60].

All H1 subtypes are rich in basic amino acids, mostly lysine. Considering that this residue is capable of acquiring different PTMs, H1 subtypes are heavily modified. Acetylation and methylation sites are quite abundant in H1. In human cell lines, acetylation sites were more abundant than methylation, while the opposite was true for mouse and human tissues [29,43]. A different study also showed that the H1 acetylation level in mESCs had increased compared to that of differentiated cells [35]. There are also differences regarding the localization of methylation and acetylation. Lysine residues in the NTD are often methylated, whereas acetylation is predominant in the GD [29,35,43].

Distinct residues of mammalian H1 subtypes have been found modified by formylation, crotonylation, ubiquitylation, citrullination, 2-hydroxyisobutyrylation, and parylation (Table S3). It can be observed in Figure 3 that most of the formylated, crotonylated, and ubiquitylated sites are located in the GD in lysine residues where other PTMs have been mapped. Thus, most lysine residues of the GD including those directly involved in DNA binding can be considered PTM-hotspots as they appear to be targeted by many different modifications, which may modulate the binding properties of this domain in response to different situations and stimuli (Figure 3).

In mammals, there are four H1 germline-specific subtypes. Several studies have characterized the H1 complement in testis, thus allowing the exploration of H1 PTMs in the male germline-specific subtypes [34,57,58,61,62] (Figure 3, Table S3). Extensive characterization of H1 PTMs has been carried out in rat testis [34,58,61]. Perchloric acid extracts were analyzed by mass spectrometry using different separation methods, capillary electrophoresis (CESI-MS), and nano-HPLC-liquid chromatography (LC-ESI-MS/MS). This procedure allowed the identification of modifications in somatic H1 subtypes H1.0–H1.5 and one of the male germline-specific subtypes, H1t. Multiple PTMs were detected in subtypes H1.1, H1.3, H1.4, and H1t, while few modified sites were found in H1.0, H1.2, and H1.5 (Table S3). Most of the sites were identified by both CE and LC, but a few were detected by only one method [34]. The most abundant PTM was phosphorylation, but a few acetylated residues were also found. Acetylation was found at the N-terminus of all the detected subtypes, and also at other positions in subtypes H1.1, H1.3, and H1t. Multiple phosphorylated sites were detected in all subtypes including most of the CDK motifs present in subtypes H1.1, H1.3, H1.4, and H1t. In agreement with mouse and human data, most of H1t PTMs were located in the CTD, but no methylated residues were detected in this species. More PTMs were detected during mouse spermatogenesis than in mature human sperm [57] (Table S3). In H1t, as in somatic subtypes, the predominant modification types included phosphorylation, methylation, and acetylation. In contrast, in H1t, the PTMs were mainly located in the CTD, whereas in somatic subtypes, PTMs have been mapped in the three structural domains of H1 (Figure 3). 

Endogenous HILS1, another male germline-specific subtype, was separated from the rest of the H1 subtypes by reversed-phase high-performance liquid chromatography (RP-HPLC), allowing the identification of 15 PTMs including acetylation and phosphorylation. In particular, phosphorylation appeared to be abundant in this protein, as over 40% of the S/T/Y residues were found phosphorylated, with most of the phosphorylation sites located in the GD (Figure 3, Figure S2, Tables S3 and S4) [58]. Interestingly, tyrosine phosphorylation was detected for the first time in linker histones at Y78 of this subtype, a residue located in the globular domain. HILS1Y78p appears in early elongating spermatids, where it co-localized with Transition Protein 2 (TP2), and disappears from the head region of condensing spermatids, remaining only in the tail region [58]. While multiple PTMs were detected in human, mouse, and rat H1t as well as in rat HILS1, no post-translational modifications have been detected in the third male-germline specific subtype, H1T2.

Summarizing, PTMs have been detected in nine out of eleven mammalian H1 subtypes, depending on the species. At least 13 modification types have been described in H1, with phosphorylation, methylation, and acetylation the most abundant. Almost 400 positions, in the consensus sequence for each subtype, have been found modified. This number is a rough estimation of the extent of modification of H1 subtypes because some residues can have alternative PTMs, and also because promiscuous peptides are mapped to more than one subtype. However, the number of PTMs whose function has been described is quite small in comparison. 

## 2. Functional Role of H1 PTMs.

Histone H1 is associated with the formation of higher-order chromatin structure. It has been described that H1 binding suppresses nucleosome unwrapping, therefore limiting chromatin accessibility [63]. The interaction of histone H1 with chromatin is mediated by basic residues. Phosphorylation and short-chain acylations (SCA) (acetylation, formylation, propionylation, and crotonylation) cause a decrease in the positive net charge of H1, which may increase its dissociation constant, favoring chromatin accessibility [64].

In core histones, methylation of histones results in either activation or repression of transcription, depending on the modified residue and the simultaneous post-translational modification of other residues [43]. In H1, methylation is related mostly to transcriptional silencing [43,65]. Methylation often occurs at the PTM-hotspots in the GD, where alternative PTM types, mostly short-chain acylations, and methylation have been mapped. Most PTM-hotspots are residues in proximity to DNA-binding sites (Figure 3, Figure S2). In this context, it has been proposed that methylation may protect the lysine residue of further modification and thus, impair chromatin relaxation induced by SCA, promoting the transition to a locally repressed chromatin state [36]. 

Twenty years ago, Strahl and Allis formulated the histone code hypothesis [66]. They proposed that distinct histone modifications acting in combination or sequentially, on one or multiple histone tails, specify unique downstream functions. During this time, much information about core histone modifications and their function has been gathered. However, due to the lack of site- and modification-specific antibodies, compared with core histones, very little is known about the histone H1 modifications, modifiers, and function [67,68]. Nevertheless, some H1 PTMs have been associated with the regulation of chromatin compaction during the cell cycle, where PTMs can favor transcriptional activation, heterochromatin formation, or disassembly [40,69,70,71]. Additionally, modifications in H1 have been linked to DNA damage response [48,72,73,74,75] as well as to differentiation [34,45,57,76,77].

### 2.1. Modifications in Histone H1 Associated with Chromatin Compaction during the Cell Cycle

Phosphorylation of linker histones in CDK-consensus motifs (S/T-P-X-K/R) has been identified in many different species [34,38,42,55,56,57]. The CDK-consensus motifs are located in the terminal domains: three or four sites in the CTD, and one site in the NTD of some subtypes. The extent of H1 phosphorylation is variable during the cell cycle. The amount of phosphorylated H1 increases from G1 to metaphase, sharply decreasing thereafter [60].

**Figure 4:**
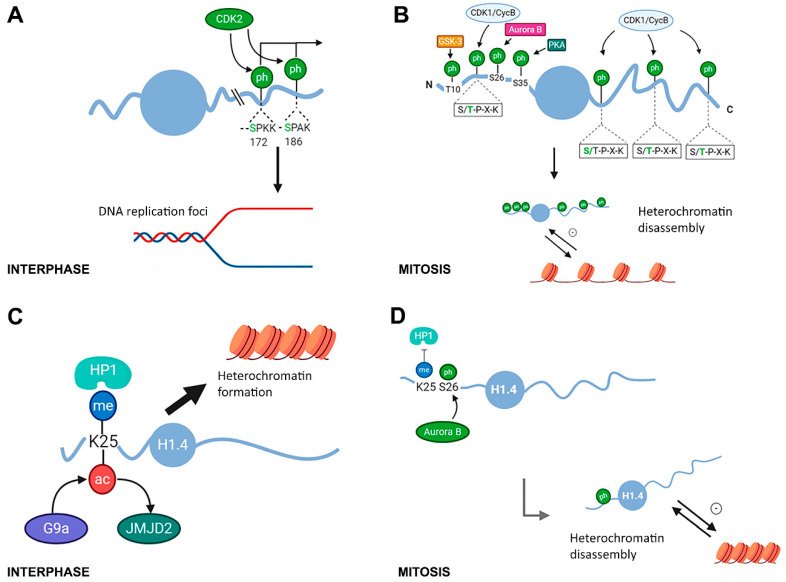
Modifications in histone H1 associated with chromatin compaction during the cell cycle. (**A**) In interphase, some CDK consensus motifs are phosphorylated presumably by CDK2. H1.2S172p and H1.4S186p have been linked to active transcription by RNAP I and II sites [40]. H1.5S172p and H1.2S172p have been shown to localize at active transcription sites and replication foci [70]. (**B**) In mitosis, H1 is hyperphosphorylated by CDK1 at CDK-consensus motifs and by GSK-3, Aurora B, and PKA at H1.5T10, H1.4S26, and H1.4S35. Those PTMs are thought to contribute to heterochromatin disassembly [69,70,71,83]. (**C**) H1.4K25 is methylated by histone methyltransferase G9a and demethylated by JMJD2 [88,89]. This modification recruits the heterochromatin protein HP1, promoting heterochromatin formation [69]. H1.K25 can also be acetylated, which would prevent methylation and HP1 binding [91]. (**D**) During mitosis, S26 is phosphorylated by the Aurora B kinase [84]. This modification inhibits HP1 binding, and thus may favor heterochromatin disassembly [69].

Histone H1 phosphorylation is a hierarchical process. During interphase, in HeLa S3 cells, H1.2S172, H1.4S171, and H1.4S186 were found to be phosphorylated [40]. In human lymphoblastic T-cells, H1.5 was first phosphorylated in S17, followed by S172 and S188 [78]. These results led to the conclusion that H1 interphase phosphorylation occurs mainly at serine residues of the CDK motifs. H1 partial phosphorylation is associated with a decrease in the residence time of H1 in chromatin and with chromatin relaxation [16,79]. Experimental evidence corroborates that H1 phosphorylation is associated with different nuclear processes that require open chromatin including transcription, replication and DNA repair [40,70,80] (Figure 4A).

Phosphorylated species are differentially distributed within the nucleus [40,70]. In Chinese hamster ovary (CHO) cells, replicating DNA colocalization in vivo with phosphorylated H1. In these cells, S-phase progression and H1 phosphorylation are directly related and dependent on Cdk2 activity [80]. In HeLa, monophosphorylated species of H1.2/H1.5 at S172, but not H1.5S17 localized to active DNA replication foci and active transcription sites [70] (Figure 4A). A second study showed that H1.2S172p and H1.4S186p are enriched in nucleoli, where H1.4S186p is preferentially associated with active rDNA promoters [40]. H1.4S186 phosphorylation occurs at hormone-responsive promoters, linking H1 phosphorylation with transcription by RNA polymerase I and II. The role and kinetics of H1 phosphorylation in transcriptional activation have been studied using the mouse mammary tumor virus (MMTV) promoter as a model system [81,82]. In this model, H1 is rapidly phosphorylated upon progesterone addition, leading to H1 eviction from chromatin and increasing the accessibility of the promoter to transcription factors. Additionally, the association of H1.4S33ac with transcriptional activation will be described below, within the context of PTMs associated with cellular differentiation.

During mitosis, CDK-consensus motifs with threonine residues become phosphorylated, accounting for most of the phosphate groups in tetra- and pentaphosphorylated H1 species [40,70]. In this phase of the cell cycle, non-CDK consensus sites H1.4S26, H1.4S35, and H1.5 T10 are phosphorylated by Aurora B kinase, protein kinase A (PKA), and glycogen synthase kinase-3 (GSK-3), respectively (Figure 4B) [71,83,84].

Hyperphosphorylation of H1 in mitosis has been associated with chromatin compaction, although, in some systems such as the amitotic macronucleus *T. thermophila* and the terminally differentiated chicken erythrocytes, chromatin condensation is uncoupled from the phosphorylation of H1 [23,38,55,68]. In mammals, treatment with staurosporine blocked H1 phosphorylation and prevented the condensation of mitotic chromosomes in CHO cells [27]. This finding was confirmed in murine cells, where the addition of staurosporine to cells blocked with nocodazole in mitosis caused rapid histone dephosphorylation and chromosome decondensation [85]. However, the function of H1 phosphorylation during mitosis is not fully understood, because the association of hyperphosphorylation with chromatin compaction is at odds with the effect of phosphorylation during interphase, where it promotes chromatin decondensation. This apparent contradiction could be explained if H1 hyperphosphorylation promoted heterochromatin disruption, which may be necessary to achieve the chromatin compaction needed in metaphase chromosomes [68]. 

Recently, it has been suggested that histone H1 can regulate chromatin organization by modulating phase separation [86,87]. It has been shown that histone H1 promoted chromatin liquid–liquid phase separation (LLPS), an effect that appeared to be mediated by its CTD [86]. Moreover, in vitro experiments with the isolated CTD showed that phosphorylation of three CDK-consensus motifs distributed along this domain reduced the presence of the CTD in micrometer-scale droplets, suggesting that phosphorylation may disperse or repartition of chromatin droplets in vivo [87]. Whether the modulation of chromatin LLPS mediated by H1 phosphorylation is relevant during interphase and/or mitosis remains to be determined. 

The studies on the methyl-phospho switch of H1.4K25-S26 have revealed how the crosstalk between PTMs in adjacent residues of H1.4 can regulate heterochromatin formation and disassembly [69] (Figure 4C,D). The NTD of human H1.4 contains an ARKS motif, which is conserved in primates and carnivora [8]. In this motif, the lysine residue is methylated in vivo by histone methyltransferase G9a and removed by the lysine demethylase JMJD2(KDM4) [88,89]. Methylation of K25 (in some references K26) within the ARKS motif recruits the heterochromatin protein HP1, promoting heterochromatin formation (Figure 4C). During mitosis, S26 is phosphorylated by Aurora B kinase [84]. This modification inhibits HP1 binding and thus, may favor heterochromatin disassembly (Figure 4D) [69]. Additionally, H1.4K25 can also be acetylated, although the enzyme responsible has not been identified. However, H1.4K25 deacetylation is mediated by Sirt1 [90]. This modification was detected alone and in combination with H1.4S26p in human breast cancer cells [91]. Acetylation of H1.4K25 would prevent methylation and HP1 binding and could also favor chromatin opening. 

Another mitosis-specific modification is the phosphorylation of H1.4S35 by PKA. It has been shown that this PTM could dissociate H1.4 from mitotic chromatin (Figure 4B). Additionally, mutations in H1.4S35 resulted in mitotic defects, underscoring the functional impact of this modification in specific mitotic functions [71]. Interestingly, this position is conserved in subtypes H1.2 and H1.3 (Figure 3, Figure S2, and Table S3), although the modification of those subtypes during mitosis has not been addressed.

The implication of other modifications in the cell cycle has been studied in humans and *Drosophila*. Phosphorylation of H1.2 at T164, a non-CDK site, accumulates during S and G2/M phase in T47D, a human breast cancer cell line. This modification was dispensable for cell proliferation and binding to chromatin [91]. In *Drosophila*, H1 dimethylated in K26 accumulates at pericentromeric heterochromatin in the metaphase, suggesting a functional contribution of this PTM to heterochromatin organization during mitosis [39].

### 2.2. Modifications in Histone H1 Associated with DNA Damage Response

Phosphorylation, acetylation, ubiquitylation, and parylation of histone H1 have been associated with DNA damage response [72,73,74,75]. Most of the examples have been described in human cell lines, but some evidence in *Saccharomyces cerevisiae* has also been found [48].

**Figure 5:**
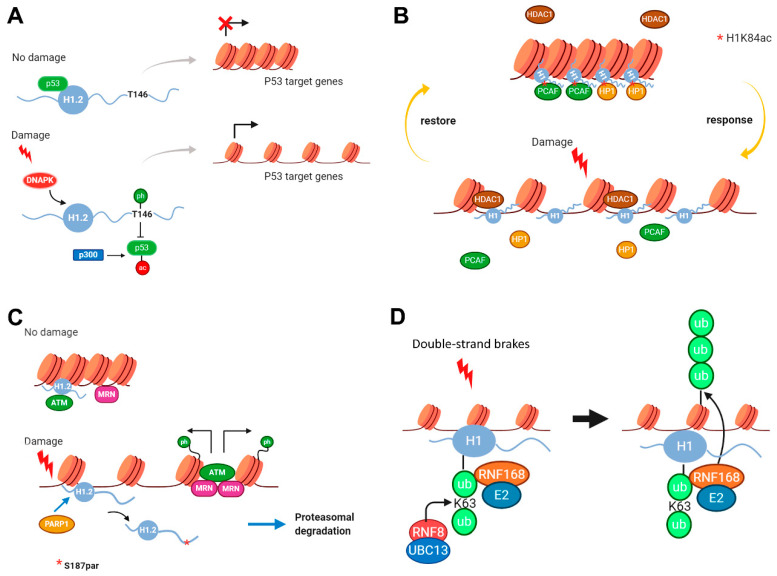
Modifications in histone H1 associated with DNA damage response. (**A**) In normal conditions, H1.2 is capable of binding p53 and maintaining p53 target genes in a quiescent state. Upon DNA damage, H1.2 is phosphorylated in T145 by DNA-PK, while p53 is acetylated by p300. These modifications disrupt p53-H1.2 interaction, allowing p53 to activate the transcription of its target genes [73]. (**B**) H1K84 is acetylated by PCAF. This modification recruits the heterochromatin protein 1 (HP1), leading to chromatin compaction. In response to DNA damage, H1K84ac rapidly decreases, removed by HDAC1. K84 deacetylation facilitates chromatin accessibility to DNA repair machinery. PCAF is gradually recruited, thereby restoring H1K84ac levels and chromatin structure after DNA repair [73,74]. (**C**) H1.2 directly interacts with the ATM, inhibiting MRN complex-dependent ATM recruitment. Upon DNA damage, H1.2S187 is parylated by PARP1, inducing its dissociation from chromatin and its degradation. H1.2 removal allows ATM activation after the recruitment of MRN complex, initiating DNA damage response through phosphorylation of different substrates, including γH2AX [74]. (**D**) In response to DNA double-strand breaks, RNF8-UBC13 catalyzes K-63 linked ubiquitylation of H1, providing an initial binding platform for RNF168, which in turn ubiquitylates H2A at K13/K15, and possibly other proteins that recruit DSB repair factors [75].

It has been shown that H1.2T145p is associated with p53-dependent DNA damage response [73] (Figure 5A). Threonine 145 in H1.2 belongs to a CDK-consensus motif, therefore in normal conditions, it becomes phosphorylated during mitosis. During interphase, unphosphorylated H1.2 is capable of binding p53 and maintaining p53 target genes in a quiescent state. Upon DNA damage, p53 is acetylated by p300 and H1.2 is phosphorylated in T145 by DNA-PK. These modifications disrupt p53-H1.2 interaction, allowing the recruitment of chromatin remodeling and transcription factors to p53 target promoters. Thus, both modifications signal the onset of the p53 transcriptional program in response to DNA damage.

Recently, it has been described that H1K84ac (referred to H1.4) is involved in DNA damage response [72] (Figure 5B). Lysine 84 is one of the PTM-hotspots of the globular domain (Figure 3). This residue is conserved in H1.1–H1.5 subtypes and is modified by acetylation, formylation, crotonylation, and 2-hydroxyisobutyrylation. More importantly, K84 is directly involved in DNA binding (Figure 3, Figure S2). Interestingly, mutation of K84 to glutamine (Q), which mimics acetylation, increased H1 binding affinity, leading to chromatin condensation. Acetylation of H1K84 also promoted the recruitment of heterochromatin protein 1 (HP1), therefore leading to chromatin compaction. ChIP-seq studies showed that H1K84ac was not enriched in promoters, confirming that it may not be involved in general transcriptional regulation. K84 is acetylated by the acetyltransferase PCAF, and this modification is removed by the histone deacetylase HDAC1. H1K84ac-mediated regulation of chromatin structure upon DNA damage is determined by the dynamics of this PTM. In response to DNA damage, H1K84ac rapidly decreases, leading to reduced H1 binding affinity to chromatin and reduced enrichment of HP1, resulting in chromatin decondensation. PCAF is gradually recruited to chromatin in response to DNA damage, thereby restoring H1K84ac levels and chromatin structure after DNA repair (Figure 5B) [72].

Parylation of H1.2 in S187 has also been linked to DNA damage response, in particular with ataxia-telangiectasia mutated (ATM) activation [74] (Figure 5C). ATM is a protein kinase, which phosphorylates several key proteins that initiate activation of the DNA damage checkpoint. It has been shown that H1.2 directly interacts with the ATM HEAT repeat domain, inhibiting MRN complex-dependent ATM recruitment. This interaction was specific, as other H1 subtypes exhibited a much weaker binding affinity to ATM than H1.2. Upon DNA damage, H1.2 is PARylated at S187 by PARP1, inducing its dissociation from chromatin and its proteasomal mediated degradation. Mutation of S187A delayed, but did not impair H1.2 displacement from chromatin, suggesting the presence of additional parylation sites. H1.2 removal allows ATM to be recruited by the MRN complex, thereby initiating DNA damage response through the phosphorylation of different substrates including γH2AX. These findings suggest that H1.2 parylation is needed for proper ATM activation.

Ubiquitylation of H1 has been associated with DNA double-strand break (DSB) repair [75] (Figure 5D). DNA double-strand breaks are cytotoxic DNA lesions that trigger non-proteolytic ubiquitylation (K63-linked) proteins in adjacent chromatin areas to generate binding sites for DNA repair factors. Two E3 ubiquitin ligases, RNF8 and RNF168, and UBC13, an E2 ubiquitin-conjugating enzyme that specifically generates K63-linked ubiquitin chains, are involved in this process. It has been shown that UBC13-dependent K63-linked ubiquitylation at DSB sites is predominantly mediated by RNF8 and that histone H1 subtypes are its major chromatin-associated targets. In fact, ubiquitylated peptides derived from the GD of H1 of all somatic subtypes were identified and the K-63 linked ubiquitylation was confirmed by pull-down in H1.2 and H1x (Table S3). Mechanistically, K-63 linked ubiquitylated H1 provides an initial binding platform for RNF168, which in turn ubiquitylates H2A at K13/K15 and possibly other proteins, to trigger the recruitment of DSB repair factors.

Finally, another example of PTMs in H1 involved in DNA damage response has been described in yeast. In Hho I, three phosphorylated residues have been characterized (Table S1). At a functional level, phospho-null mutations S➔A have shown that phosphorylation of two of those residues, S172 and S173, was required for double-strand break (DSB) repair by homologous recombination (HR). Presumably, Hho I phosphorylation at S172 and S173 increases chromatin accessibility, facilitating DSB repair by HR [48].

### 2.3. Histone H1 PTMs in Cell Differentiation and Aging

**Figure 6:**
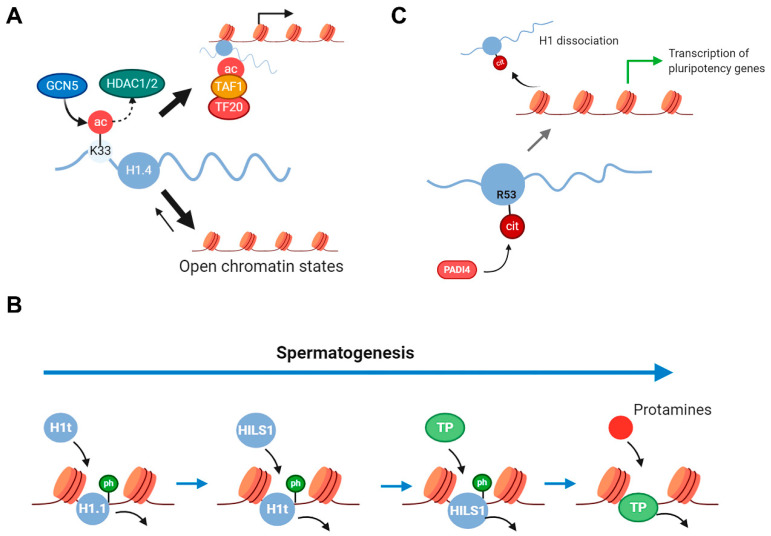
Modifications in histone H1 associated with cell differentiation. (**A**) H1.4K33 acetylation is catalyzed by GNC5 and can be deacetylated by class I and class II HDACs H1.4K33ac promotes transcriptional activation by two different mechanisms: by reducing H1.4 affinity for chromatin and by recruiting TAF1 [78]. (**B**) During spermatogenesis, male germ cells undergo a unique chromatin remodeling process characterized by the sequential substitution of somatic H1 (the most abundant subtype is H1.1), by testis-specific subtypes (H1t and HILS1), transition proteins (TP), and protamines. The detection of multiple phosphorylated positions in H1.1, H1t, and HILS1 in rat testis, during mouse spermiogenesis, and in human sperm, suggests that phosphorylation of H1 subtypes facilitates protein substitution throughout spermatogenesis [34,57,58]. (**C**) Citrullination of R53 (referred to H1.2) by PADI4, a residue located in the DNA-binding site of the GD of H1, results in its displacement from chromatin and global chromatin decondensation, which is required for the transcriptional activation of pluripotency genes [45].

Several post-translational modification types in H1 have been associated with cell differentiation including acetylation, phosphorylation, and citrullination (Figure 6). H1.4K33 acetylation is catalyzed by GNC5 and can be deacetylated by class I and class II HDACs, but not by sirtuins [76]. ChIP-seq analysis showed that H1K33ac was enriched at active promoters, where it colocalized with H3K4me3. H1.4K33ac increases H1 mobility and is associated with transcriptional activation. This PTM promotes transcriptional activation by two different mechanisms: by reducing H1.4 affinity for chromatin and by recruiting TAF1, a subunit of the general transcription factor TFIID (Figure 6A). This modification is upregulated in induced pluripotent stem (iPS) cells, favoring H1 mobility, and the generation of the open chromatin state, characteristic of pluripotent stem cells. The dynamic behavior of this modification during spermatogenesis has also been studied, where high levels of H1.4K33ac were observed in the elongating spermatids, coinciding with chromatin reorganization. However, the signal disappeared in condensing spermatids and spermatozoa, suggesting that this PTM could contribute to chromatin opening, and therefore to the exchange of H1 with the testis-specific subtypes [76].

Several H1 PTMs have been identified in testis that could be important for spermatogenesis [34,57,58] (Figure 6B). Spermatogenesis is a differentiation process that can be divided into three phases: (1) stem cell renewal and differentiation, (2) meiosis, and (3) spermiogenesis. During mammalian spermatogenesis, male germ cells undergo a unique chromatin remodeling process, which results in a highly compact and condensed chromatin structure in mature sperm. In this process, somatic histones are replaced, sequentially, by testis-specific variants, transition proteins, and finally, by protamines [92].

Among somatic H1 subtypes, H1.1 is the predominant H1 variant in pre-pachytene spermatocytes, comprising approximately 70% of the total H1 [59]. As previously mentioned, there are three male germ-line specific H1 subtypes: H1t, HILS1, and H1T2. Histone H1t is detected from mid-pachytene spermatocytes until the elongating spermatid stage and replaces about 40% of H1 somatic subtypes. HILS1 is detected later in elongating and condensing spermatids nucleus, while H1T2 is implicated in the replacement of histones by protamines [92]. Histone H1 PTMs during mouse spermiogenesis, in rat testis and human sperm have been characterized (Figure 3, Table S3). In rat testis, 11, 10, and 11 phosphorylated residues were detected in H1.1, H1t and HILS1, respectively [34,57,58]. During mouse spermiogenesis, eight phosphorylations were found in H1t, while only two phosphorylations were detected in H1t from human sperm [57]. In this context, H1 phosphorylation would contribute to the formation of loosely compacted chromatin states necessary for genetic recombination in pachytene spermatocytes, and for the replacement of histones with transition proteins in early spermatids.

Histone H1 arginine citrullination has been associated with pluripotency [45] (Figure 6C). Citrullination is an irreversible PTM in which arginine is deimidated to citrulline. The enzymes responsible for this modification are called peptidylarginine deiminases (PADIs). PADI4 participates in the pluripotency transcriptional network by activating the expression of key-stem cell genes. Inhibition of PADI4 lowered the percentage of pluripotent cells in the early mouse embryo and significantly reduced reprogramming efficiency. Proteomic analysis of mESCs identified subtypes H1.2, H1.3, and H1.4 as PADI4 targets. Citrullination of a single arginine residue, R53 (referred to H1.2), within the DNA-binding site of the GD of H1 subtypes results in its displacement from chromatin and global chromatin decondensation. This finding suggests the involvement of PADI4-induced citrullination of H1 in the regulation of pluripotency. Citrullination at the equivalent position in human H1 subtypes has also been identified in breast cancer cells [37].

The key similarities between aging and differentiation are the reorganization of eu- and heterochromatic domains, and an increase in heterochromatin, known as heterochromatinization. Accumulation of H1.0 has been described in an in vitro aging system of human diploid fibroblasts [93]. Additionally, it has been found that H1.0 undergoes age-dependent deamidation [94]. An increase in H1.0 deamidated in asparagine 3 and N-terminally acetylated was found in humans, mice, and rats upon aging. Histone H1.4 and H1.5 PTMs have also been associated with aging. Analysis by capillary zone electrophoresis (CZE) has shown that dephosphorylation of H1.4 and H1.5 correlated with an increase in senescence-associated heterochromatin formation during in vivo aging of human peripheral blood lymphocytes [79].

## 3. Histone H1 PTMs Associated with Disease

Post-translational modifications in histone H1 have been associated with cancer, autoimmune diseases, and viral infection [37,95,96,97,98,99,100,101,102,103]. Different modification types in H1 have been described in disease including, phosphorylation, methylation, ubiquitylation, and citrullination.

PTMs associated with cancer have been extensively studied to find new biomarkers of cancer progression and prognosis to personalize therapeutic strategies as well as to understand the underlying mechanisms of the disease. Several phosphorylations in subtypes H1.2–H1.5 have been associated with cancer progression and prognosis [37,95,96,97,98,101,103]. Phosphorylation of H1.2 and H1.4 at T145 has been proposed as a biomarker for bladder cancer [95]. MS profiling revealed a statistically significant increase in phosphorylation of H1.2 and H1.4 from normal human bladder epithelial cells to low-grade superficial to high-grade invasive bladder cancer cells. Interestingly, H1.3 phosphorylation was not significantly increased, despite sharing the same consensus motif with H1.2 and H1.4. Phosphorylation of H1.5 was increased, but the phosphorylated positions were not characterized. In human bladder cancer samples, the percentage of positive H1.4T145p staining correlated with an increase in the histopathologic grade, invasiveness, and proliferation rate, suggesting that these PTMs could also reflect cancer progression [95].

The same residue in H1.2 is phosphorylated in response to DNA damage by DNA-PK [75]. Very recently, it has been shown that metastasis-associated 1 (MTA1) inhibited H1.2T145 phosphorylation by mediating proteasomal degradation of DNA-PK [97]. This inhibition was associated with tumorigenesis and metastatic progression in hepatocellular carcinoma (HCC). Moreover, MTA1’s oncogenic role was inhibited by ectopic expression of H1.2T145p in HCC cell lines, underscoring the importance of H1.2T145 phosphorylation in the therapeutic axis in HCC.

Proteomic profiling of H1 PTMs in breast cancer cell lines identified a tyrosine residue phosphorylated Y70 (referred to H1.2) in H1.2, H1.3, and H1.5 [37]. This modification is located in the GD, in a region very conserved in human H1.1–H1.5 subtypes. However, the previous separation of H1 subtypes only detected H1.2, H1.3, and H1.5. Sequence analysis of the phosphosite suggested that FAK kinase could be the enzyme to catalyze this modification. The role of FAK in the catalysis of Y70 phosphorylation was supported by pharmacological inhibition of this kinase, co-immunoprecipitation, and co-localization experiments. The levels of tyrosine phosphorylation in H1 subtypes were significantly higher in breast cancer cells when compared to normal cells, suggesting a role of this modification in breast cancer. It was also established that H1 tyrosine phosphorylation positively correlated with the cell-proliferative status, suggesting a role of H1Y70p in the definition of the tumor phenotype [37].

Ras mutation and ERK activation appear in most human cancers. It has been shown that Ras mutations repressed H1.4S35p by inducing the degradation of the writer of this PTM, PKA, in an MDM2-dependent way in non-small-cell lung carcinoma (NSCLC) cell lines [96]. H1.4S35p showed a tumor suppressor role as the overexpression of a mutant mimicking phosphorylation attenuated cell viability, colony formation, S-phase arrest, migration, and invasion upon mutant Ras transfection in NSCLC cell lines. A similar effect was found for the phosphorylation of H1.4 in S26 in gastric cancer cells (SNU-16), where H1.4S26p reduced cell viability, colony formation, and migration [103]. Ras mutation, accompanied by ERK1/2 activation, repressed H1.4 phosphorylation at S26 by the induction of MDM2-dependent proteasomal degradation of Aurora B.

In addition to phosphorylation, H1 methylation, and in particular K84 monomethylation, has also been associated with cancer, specifically with squamous cell carcinoma of the head and neck (SCCHN) cells, a malignancy with poor outcome [98]. This PTM is catalyzed by the protein methyltransferase WHSC1, which is known to dimethylate lysine 36 of histone H3. Lysine 84 is located in a highly conserved region of the globular domain, suggesting that this modification could be present in other subtypes. Furthermore, the role of the acetylation in K84 in response to DNA damage has already been discussed [74]. MS analysis showed that this methylase could interact with different H1 subtypes, and in vitro methylation of several subtypes was confirmed. However, for the functional studies, the authors focused on H1.4. WHSC1 is significantly overexpressed in SCCHN cells, where H1.4K84me1 has been described to induce changes in the gene expression of more than 400 genes. The most relevant finding was the change in the expression of Oct4 and several of its target genes, suggesting that WHSC1-mediated H1.4K84me1 may contribute to the maintenance of cancer stemness features in SCCHN cells.

Several PTMs in H1.2, H1.3, H1.4, and H1.5 were identified in tissue samples of pancreatic ductal adenocarcinoma (PDAC) [101]. One ubiquitylated and two monomethylated residues were found in H1.2. In H1.3 and H1.5, two and three ubiquitylated sites were detected, respectively. The subtype with more PTMs was H1.4, with eight residues methylated in the NTD and the GD, and one residue ubiquitylated. The presence of these modifications was sporadic in the cohort analyzed and their role in cancer progression was not explored.

In addition to its role in pluripotency, citrullination of H1R53 is associated with autoimmune disorders such as systemic lupus erythematosus (SLE) [99]. Neutrophil extracellular traps (NET) formation (or NETosis) is of key importance as a first-line defense against bacteria, viruses, and protozoa. Several autoimmune diseases seem to share these pathogenic mechanisms, including SLE. In these diseases, NETosis is induced by inflammatory conditions. In this process, histone citrullinated peptides are released to the bloodstream, where they induce the formation of autoantibodies. Citrullinated peptides containing H1R53cit, H1.0R73cit, and H1.0R93cit were detected in activated neutrophils. Evaluation of the sera from SLE patients found reactivity in a small subset of patients against an H1.2 citrullinated peptide. However, the citrullinated peptides of H1.0 were not assayed. Very recently, sera from patients of coronavirus disease 19 (COVID-19) have shown high levels of NETosis-specific markers including H3 citrullinated peptides [104]. This finding suggests that H1 citrullinated peptides may also be present in these patients. Further research is needed to determine the predictive value of circulating citrullinated peptides in the outcome of the disease and if NETs can provide an additional therapeutic target against COVID-19.

Histone H1 PTMs have also been associated with viral infection [100,102]. Herpes simplex virus (HSV-1) infection leads to changes in host chromatin and proteome. One of the modifications found upon HSV infection was an increase of H1.4K25 dimethylation, concomitant with a decrease in the phosphorylation of the adjacent residue H1.4S26 [102]. Interestingly, these modifications are in the ARKS motif of H1.4, where a methyl-phospho switch, similar to that of H3K9-S10 has been described [69]. The changes in the PTMs of the H1.4 methyl-phospho switch were opposite to those found in the H3 methyl-phospho switch, which have been associated with HSV-1 reactivation from latency [105].

Monoubiquitylated histone H1.5 has been associated with antiviral protection in CD4+T cells resistant to HIV-1 [100]. Some human cell lines are resistant to HIV-1 infection and secrete a soluble factor, named HIV-1 resistance factor (HRF), capable of inhibiting HIV-1 replication in susceptible cells. Monoubiquitylated H1.5 coeluted with HRF. Although H1.5 was not required for HRF-mediated antiviral protection in HIV-1 target cells, its expression was indispensable for HRF activity in both intra- and extracellular compartments. It was hypothesized that the monoubiquitylation of H1.5 somehow contributed to the expression of HRF and that the subsequent secretion of H1.5ub1 into the extracellular compartment might also facilitate the transport of HRF [100].

## 4. Future Perspectives and Challenges

In the last 15 years, significant advances have been made regarding the identification of PTMs in histone H1. We envision that in the next few years, research on histone H1 PTMs will be focused on four main aspects. The first, and most obvious, is the identification of the PTMs in subtypes, H1x, H1T2, and H1oo, for which very little information is known. H1T2 and H1oo are restricted to germinal cells, but H1x is widely expressed, albeit at low levels. Thus, a purification step separating H1x from the rest of the H1 subtypes, before MS analysis would be useful.

The second aspect concerns PTM quantification. Relative quantification of H1 PTMs based on different label-free approaches or based on the metabolic labeling of the proteins has been carried out [43,55,77,95]. Nevertheless, systematic relative and absolute quantification of H1 PTMs in health and disease is needed. For relative quantification in cell culture, stable isotope labeling by amino acids in cell culture (SILAC) has been widely used. However, for clinical samples, an alternative would be the use of isobaric labeling, where using tandem mass tags (TMT), up to 16 samples could be analyzed in parallel. For absolute quantification of core histones, a modified SILAC method has been used [106]. Another option would be approaches based on the addition of isotopically-labeled peptides (Selected Reaction Monitoring (SRM), Parallel Reaction Monitoring (PRM), or Multiple Reaction Monitoring (MRM)), in which the quantification of specific modifications could be coupled with the determination of each subtype abundance using subtype-specific peptides. This technique has already been used successfully for PTM quantification in core histones [107].

The third aspect would be to characterize H1 proteoforms, which will allow assigning PTMs present in promiscuous peptides to individual subtypes, and to determine H1 PTMs coexisting in the same protein molecule [108]. The top-down experiments needed to achieve this objective would be quite challenging due to the high number of PTMs in H1, to the presence of multiple subtypes with high sequence identity, and to the complexity and low sensitivity of the analysis. Therefore, top-down approaches might be complemented with a bottom-up analysis. Alternatively, middle-down analysis could offer information of coexistent PTMs with higher sensitivity and more simple spectra than top-down analysis [109].

The final aspect would be to increase the current knowledge of the functional role of histone H1 PTMs, their integration in the histone code, and their alterations in disease. It would be necessary to find a way to overcome the lack of specific antibodies to accomplish this goal. Especially relevant is the profiling of H1 PTMs in disease. Evidence gathered from core histone PTMs have shown that patient samples and cell lines had a different PTM profile, underscoring the need to characterize PTMs from clinical samples [110]. For high-throughput studies in clinical samples including paired tissue tumor samples, approaches based on isobaric labeling, MALDI imaging, or the recently developed direct-injection mass spectrometry (DI-MS) [111] could be used. Analysis of PTMs from patient samples instead of cultured cell lines would potentially uncover new PTM marks associated with disease.

## Supplementary Materials

Supplementary materials can be found at https://www.mdpi.com/1422-0067/21/16/5941/s1.
