# Supplementary figures and images for: Histone H1 Post-Translational Modifications: Update and Future Perspectives
Source: Int J Mol Sci. 2020 Aug 18;21(16):5941. doi: 10.3390/ijms21165941 (PMC7460583; doi:10.3390/ijms21165941)

A

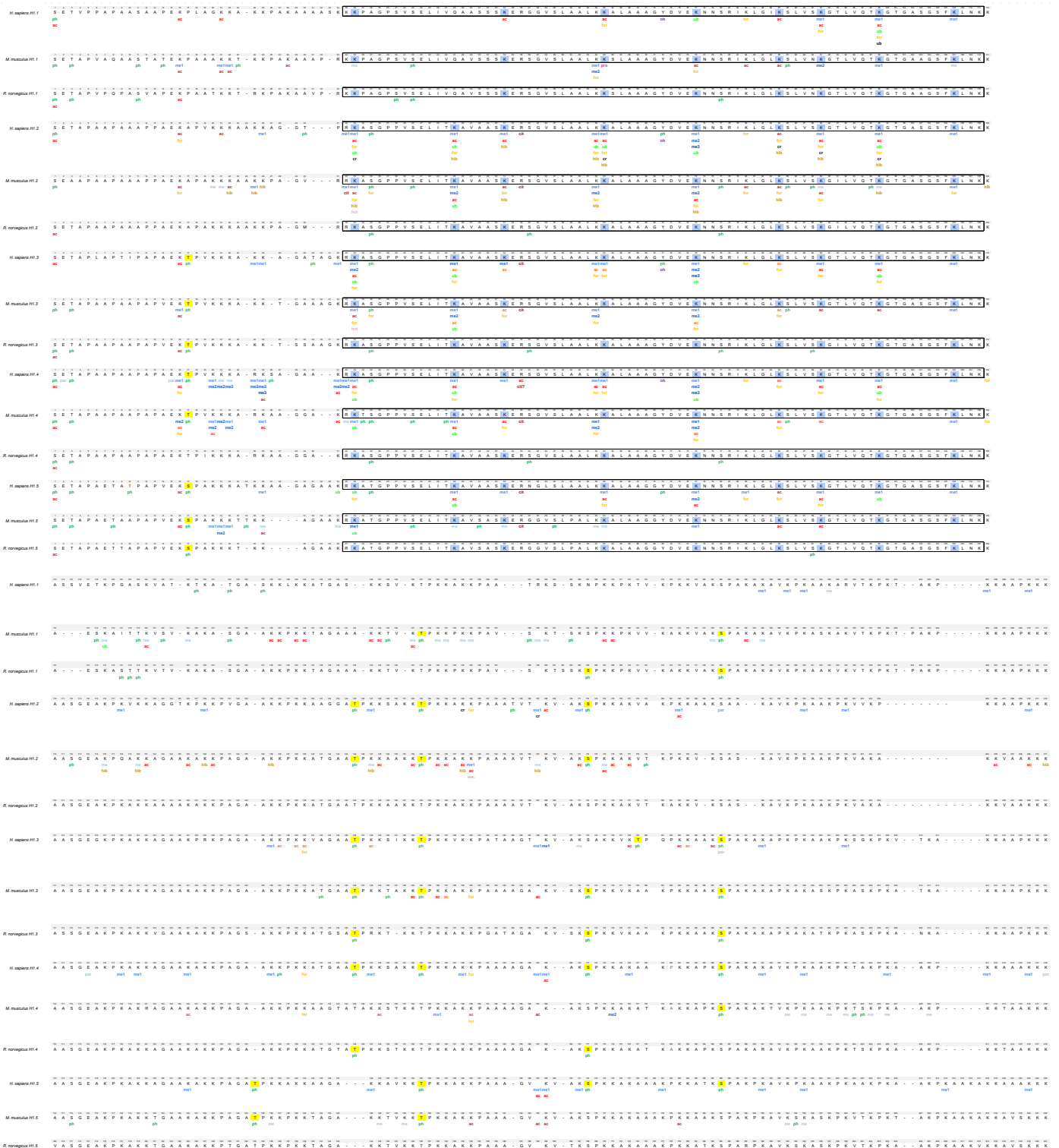

B

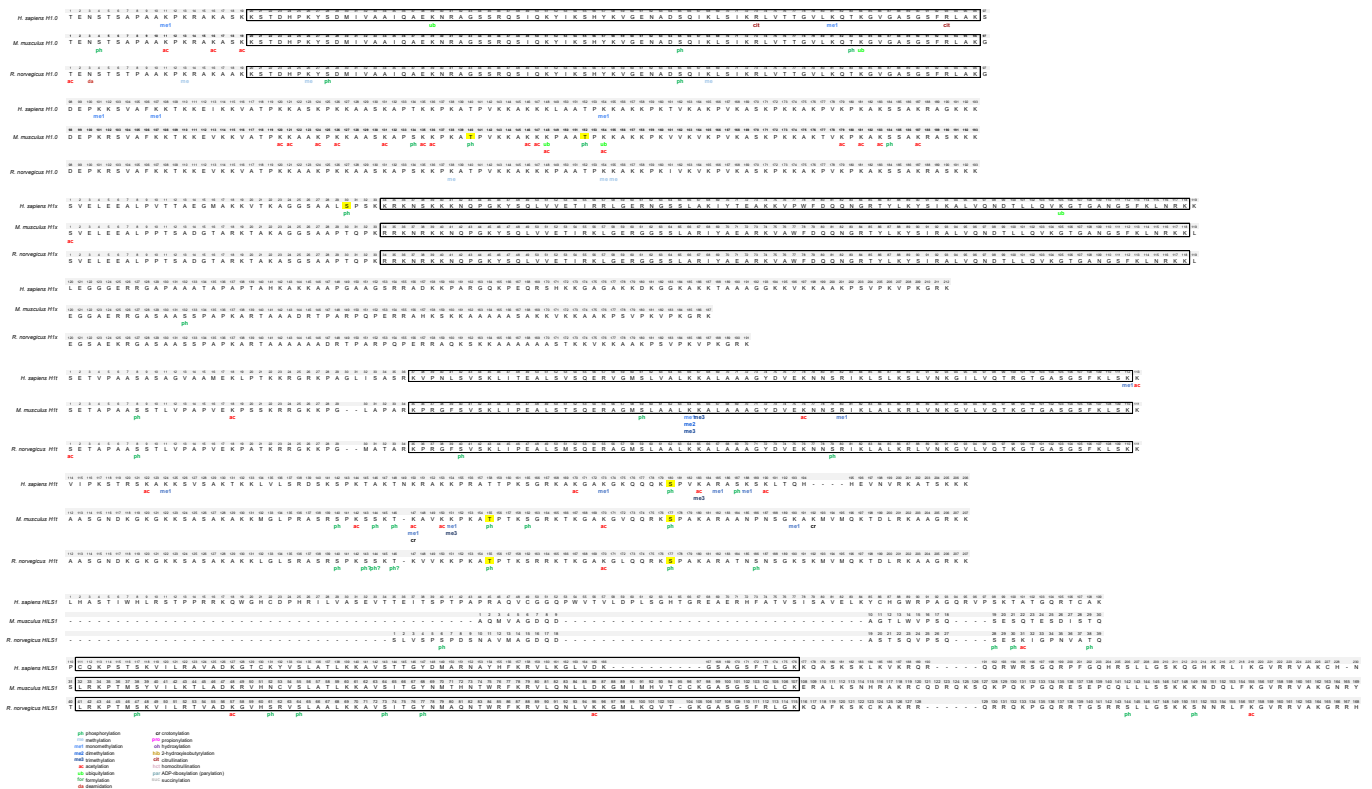

Supplement: Supplementary file 1 [file ijms-21-05941-s001.zip › Figure S2.pdf]
